# ﻿Nomenclatural notes and typification of three synonyms of *Camellia* (Theaceae)

**DOI:** 10.3897/phytokeys.193.78810

**Published:** 2022-03-15

**Authors:** Dongwei Zhao

**Affiliations:** 1 Department of Forestry, College of Forestry, Central South University of Forestry and Technology, 498 Shaoshan South Road, Changsha, Hunan 410004, China Central South University of Forestry and Technology Changsha China

**Keywords:** *Camelliacuspidata*, *Camelliareticulata*, *Camelliastrum*, *Desmitus*, lectotypification, neotypification, *Salceda*, *Theopsis*

## Abstract

All the known generic synonyms of *Camellia* are summarized with their types clarified. *Camelliastrum* and *Desmitus* are lectotypified and *Salceda* is neotypified. “*Kailosocarpus*” and “*Parapiquetia*” were not validly published, *Theaphylla* and *Tsia* are illegitimate replacement names for *Thea*, and *Kemelia* and *Tsubaki* are illegitimate replacement names for *Camellia*. Nomenclatural notes on *Theopsis* and its type are also provided.

## ﻿Introduction

Linnaeus (1753: 698) established the monotypic genus *Camellia* L. for the ornamental species, *Camelliajaponica* L. He referenced Kaempfer’s (1712: 850, 852) description and a single illustration (Kaempfer 1712: t. 851) under *C.japonica* without citing any specimens in the protologue (Arts. 38.1, 38.5, 38.6 & 38.13 of the Shenzhen Code, Turland et al. 2018; hereafter ICN). Subsequently, Bartholomew (Jarvis et al. 1993: 29) designated the illustration (Kaempfer 1712: t. 851) as the lectotype of *C.japonica* (Art. 9 Ex. 2 of the ICN). Accordingly, this illustration serves as the type of *Camellia*.

The boundaries of *Camellia* have been gradually enlarged by several taxonomists (e.g., Sweet 1818; Seemann 1859; Sealy 1958; Chang 1981; Ming 2000). Zhang et al. (2014) argued that *Camellia* might be a paraphyletic group in their analyses using four plastid DNA regions and the paralogous nuclear *LEAFY* marker. However, the monophyly of the genus was supported by investigations using complete plastid genome data (Yu et al. 2017) and other nuclear sequences (Vijayan et al. 2009; Li et al. 2011; Zhao et al. 2022). Nevertheless, nomenclatural problems should be resolved before a comprehensive phylogenetic study of the boundaries of *Camellia*. Although the genus, including tea, camellias and oil camellias, has been revised several times (e.g., Sealy 1958; Chang 1981; Ming 2000), the types of many taxa in *Camellia* have only recently been clarified (Zhao et al. 2017a, b, 2018, 2019; Zhao 2021). However, the types of some synonyms at generic rank remain unclear and are discussed here.

## ﻿Materials and methods

Relevant collections or their images from herbaria at A, BM, E, G, IBK, IBSC, K, KUN, L, LINN, P, PE, SBT, SYS, TCD, UPS and US (acronyms following Thiers 2022), and the taxonomic literature were examined. Types were chosen based on Arts. 9 and 10 of the ICN.

## ﻿Types of the synonyms of *Camellia*

Since the latest monograph of *Camellia* (Ming 2000), two further genera, *Bembiciopsis* H. Perrier (Judd 1997) and *Dankia* Gagnep. (Hô 1991; Zhao et al. 2017b), have been placed into the synonymy of *Camellia*. All 18 synonyms of *Camellia* (Linnaeus 1753; Adanson 1763; Blume 1825; Siebold 1832; Rafinesque 1830, 1838; Blanco 1845; Hallier 1921; Gagnepain 1939; Nakai 1940; Perrier 1940; Hu 1956, 1965) and their types (Jarvis et al. 1993; Lin et al. 2008; Zhao et al. 2016, 2017b, 2018, 2019; Zhao 2021) are listed in Table 1. “*Kailosocarpus*” (Hu 1957: 170) and “*Parapiquetia*” (Hu 1957: 170) were cited by Chang (1981: 12) in the synonymy of *Camellia*; however, both “*Kailosocarpus*” and “*Parapiquetia*” were not validly published since a Latin description or diagnosis was neither provided nor cited in each of the protologues (Art. 39.1 of the ICN). “*Kailosocarpus*” and “*Parapiquetia*” have no status under Art. 12.1 of the ICN and are, therefore, excluded from Table 1. The typification and nomenclatural notes of some synonyms of *Camellia* are elaborated below.

**Table 1.** A summary of synonyms of *Camellia* L.

Synonym	Type, as species (basionym)	Type, as specimen or illustration	Notes
*Bembiciopsis* H. Perrier, Mém. Mus. Natl. Hist. Nat. 13: 300. (1940)	*Bembiciopsisuniflora* H. Perrier	*Le Myre de Vilers s.n.*, P00389083, holotype	
*Calpandria* Blume, Bijdr. fl. Ned. Ind. 178. (1825)	*Calpandrialanceolata* Blume	L 0064294, lectotype (designated by Zhao et al. 2019: 298)	
*Camelliastrum* Nakai, J. Jap. Bot. 16: 699. (1940)	*Camelliacaudata* Wall.	*H. Bruce s.n.* in *Wallich 978* (K001110475, right-hand specimen), lectotype (designated by Zhao et al. 2017b: 172)	
*Dankia* Gagnep. in Humbert, Fl. Indo-Chine, Suppl. 1: 198. (1939)	*Dankialangbianensis* Gagnep.	*Poilane 18648* (P00754831), lectotype (designated by Zhao et al. 2017b: 173)	
*Desmitus* Raf., Sylva Tellur. 139. (1838)	*Camelliareticulata* Lindl.	Lindl., Bot. Reg. 13: t. 1078 (1827), lectotype, designated in this paper	
*Drupifera* Raf., Sylva Tellur. 140. (1838)	*Theaoleosa* Lour.	*Loureiro s.n.* (P00150891), holotype	
*Glyptocarpa* Hu, Acta Phytotax. Sin. 10: 25. (1965)	*Pyrenariacamellioides* Hu	*Wang 72468* (PE 00024548), lectotype (designated by Lin et al. 2008: 1701)	
*Kemelia* Raf., Sylva Tellur. 139. (1838)	*Camelliajaponica* L.	Kaempfer, Amoen. Exot. Fasc. t. 851. (1712), lectotype	Illegitimate replacement name for *Camellia*
*Piquetia* (Pierre) Hallier f., Beih. Bot. Centralbl. 39(2): 162. (1921)	*Theapiquetiana* Pierre	*Pierre 1708* (P01903371), lectotype (designated by Zhao et al. 2018: 94)	
*Salceda* Blanco, Fl. Filip. ed. 2: 374. (1845)	*Salcedamontana* Blanco	*Ramos & Edaño 34071* (K), neotype, designated in this paper	
*Sasanqua* T. Ness in Siebold, Nippon 2(6): 13. (1832)	*Camelliasasanqua* Thunb.	*Thunberg s.n.* (UPS No. 16143, left-hand specimen), lectotype (designated by Zhao 2021: 297)	
*Stereocarpus* (Pierre) Hallier f., Beih. Bot. Centralbl. 39(2): 162. (1921)	*Theadormoyana*	The author and typification of the species is dependent on a binding decision to be made, see Zhao et al. (2016: 1183)	
*Thea* L., Sp. Pl. 515. (1753)	*Theasinensis* L.	Kaempfer, Amoen. Exot. Fasc. 606, f. 1–2. (1712), lectotype (designated by Bartholomew in Jarvis et al. 1993: 93)	
*Theaphylla* Raf., Med. Fl. 2: 267. (1830)	*Theasinensis* L.	Kaempfer, Amoen. Exot. Fasc. 606, f. 1–2. (1712), lectotype	Illegitimate replacement name for *Thea*
*Theopsis* (Cohen-Stuart) Nakai, J. Jap. Bot. 16: 704. (1940)	*Theacuspidata* Kochs	*Henry 7026* (K000380525), lectotype (designated by Sealy 1958: 57)	
*Tsia* Adans., Fam. Pl. 2: 450. (1763)	*Theasinensis* L.	Kaempfer, Amoen. Exot. Fasc. 606, f. 1–2. 1712, lectotype	Illegitimate replacement name for *Thea*
*Tsubaki* Adans., Fam. Pl. 2: 399. (1763)	*Camelliajaponica* L.	Kaempfer, Amoen. Exot. Fasc. t. 851. 1712, lectotype	Illegitimate replacement name for *Camellia*
*Yunnanea* Hu, Acta Phytotax. Sin. 5: 282. (1956)	*Yunnaneaxylocarpa* Hu	*Yu 16021* (PE 00133872), lectotype (designated by Lin et al. 2008: 1702)	

### ﻿1. Camelliastrum

Taxon classificationPlantaeEricalesTheaceae

Nakai, J. Jap. Bot. 16: 699. (1940)

98C32C99-C667-53EA-9D3C-20632C2D9E35

Table 1

#### Type

(“lectotype”, Art. 10 Note 1 of the ICN; designated here): *Camelliastrumcaudatum* (Wall.) Nakai.

#### Nomenclatural notes.

Nakai (1940) established the genus *Camelliastrum* to categorize six species in China and Japan, including *Camelliastrumassimile* (Champ. ex Benth.) Nakai, *Camelliastrumbuisanense* (Sasaki) Nakai, *Camelliastrumcaudatum*, *Camelliastrumgracile* (Hemsl.) Nakai, *Camelliastrummairei* (H.Lév.) Nakai and *Camelliastrumsalicifolium* (Champ.) Nakai, but did not designate a type. Based on the description in the protologue, *Camelliastrumcaudatum* is selected as the type of the genus. The basionym of *Camelliastrumcaudatum*, *Camelliacaudata* Wall., was lectotypified by Zhao et al. (2017b: 172) as the specimen *H. Bruce s.n.* in *Wallich 978* (right-hand specimen of K001110475. An image is available at http://www.kew.org/herbcatimg/680707.jpg). Therefore, this specimen becomes the type of *Camelliastrum* (Art. 10.1 of the ICN).

### ﻿2. Desmitus

Taxon classificationPlantaeEricalesTheaceae

Raf., Sylva Tellur. 139. (1838)

1364F261-B2CA-57A6-950F-D3DEB09EA5B0

Table 1

#### Type.

*Desmitusreticulata* (Lindl.) Raf. ≡ *Camelliareticulata* Lindl., Bot. Reg. 13: t. 1078 (1827).

#### Lectotype

(designated here): Lindl., Bot. Reg. 13: t. 1078 (1827).

#### Nomenclatural notes.

Rafinesque (1838) established the monotypic genus *Desmitus* for *D.reticulata*. This was a new combination based on *C.reticulata* because he referenced the basionym by the words “Camel. do bot. reg. 1978...”. However, the taxon number cited by Rafinesque (1838: 140) is incorrect, but should be recognized as a correctable typographical error of “1078” and so does not prohibit the valid publication of the new combination (Art. 41.3 of the ICN). Nevertheless, Lindley (1827: t. 1078) described *C.reticulata* based on the living plants that bore semidouble flowers and introduced from China. No specimen was cited in the protologue of *C.reticulata*. The coloured drawing, t. 1078, was accompanied by the protologue and therefore designated as the lectotype of *C.reticulata*. Accordingly, the drawing serves as the type of *Desmitus* (Art. 10.1 of the ICN).

### ﻿3. Salceda

Taxon classificationPlantaeEricalesTheaceae

Blanco, Fl. Filip. ed. 2: 374. (1845)

3F90E0B0-B5E4-5C14-89F4-081176700977

Table 1

#### Type.

*Salcedamontana* Blanco

#### Neotype

(designated here): Philippines. Luzon: Bulacan, Angat, February 1919, *Ramos & Edaño 34071* (K!; isoneotypes: BM!, P04511451 [the image is available at https://science.mnhn.fr/institution/mnhn/collection/p/item/p04511451]!, and US 00113902 [the image is available at http://n2t.net/ark:/65665/3e093c26d-7aa1-4494-8723-989167baf8ba]!).

#### Nomenclatural notes.

Blanco (1845: 374) established the monotypic genus *Salceda* for *S.montana*. Merrill (1905: 21) transferred the species to *Thea* as *T.montana* (Blanco) Merr. and stated that “Blanco’s specimens were from Angat, Province of Bulacan”. However, the types of Blanco’s species were suggested to be either all destroyed (Merrill 1905: 6) or no longer extant (Merrill 1918: 5). I also failed to find the original material of *S.montana*. Based on the protologue (Blanco 1845: 374), a specimen collected from the same locality, *Ramos & Edaño 34071* (K), is designated as the neotype of *S.montana* because it bears flower fragments and seeds on the sheet.

##### Illegitimate replacement names for *Camellia* and *Thea*

*Thea* L., a genus established by Linnaeus (1753: 515) for tea (*T.sinensis* L., currently *C.sinensis* [L.] Kuntze), was treated as a synonym of *Camellia* by Sweet (1818: 157). *Theaphylla* Raf. and *Tsia* Adans. are illegitimate replacement names for *Thea* because *Thea* was cited in the synonymies of them (Adanson 1763: 613; Rafinesque 1830: 267), which makes *Tsia* and *Theaphylla* nomenclaturally superfluous (Arts. 6.11, 52.1 & 52.2[e] of the ICN). Similarly, *Kemelia* Raf. and *Tsubaki* Adans. are illegitimate replacement names for *Camellia* (Adanson 1763: 399; Rafinesque 1838: 139) because *Camellia* was cited as a synonym of them (Arts. 6.11, 52.1 & 52.2[e] of the ICN). Therefore, the four names, *Kemelia*, *Theaphylla*, *Tsia* and *Tsubaki*, are rejected under Art. 52.1 of the ICN.

## ﻿Nomenclatural notes on *Theopsis*

Cohen-Stuart (1916: 70) established Camelliasect.Theopsis Cohen-Stuart in his Ph.D. thesis, which constituted an effective publication under Art. 30.9 of the ICN. Subsequently, he translated the first two chapters of his original thesis in Dutch into English and published it (Cohen-Stuart 1919). Cohen-Stuart (1916, 1919) listed nine species, viz. *C.costei* H. Lév., *C.cuspidata* (Kochs) hort., *C.euryoides* Lindl., *C.forrestii* (Diels) Cohen-Stuart, *C.henryana* Cohen-Stuart, *C.lutchuensis* T. Itô ex T. Itô & Matsum., *C.parvifolia* (Hayata) Cohen-Stuart, *C.punctata* (Kochs) Cohen-Stuart and *C.rosiflora* Hook., in the key under sect. Theopsis. These nine species are presumably treated as the members of sect. Theopsis based on the structure of the key, the description of the section and the discussion. However, Cohen-Stuart (1916, 1919) did not designate a type for his sect. Theopsis. Nakai (1940) raised this section to generic rank to include 14 species and cited Cohen-Stuart’s (1919) English article (Arts. 41.1 & 41.3 of the ICN), but without selecting a type for the genus. Remarkably, four of nine species of Cohen-Stuart’s (1916, 1919) sect. Theopsis were excluded and five of them, including *C.euryoides*, *C.forrestii*, *C.lutchuensis*, *C.parvifolia*, and *C.rosiflora* (= *C.maliflora*, according to Cohen-Stuart 1916: 69, 1919: 241), were retained in the genus *Theopsis* (Cohen-Stuart) Nakai. Later, Sealy (1958: 14) treated *Theopsis* as a synonym of *Camellia* and resumed sect. Theopsis (Sealy 1958: 48). Chang (1981: 128) followed Sealy’s (1958) treatment and designated *C.cuspidata*, a species excluded from Nakai’s genus *Theopsis*, as the type of sect. Theopsis and this typification must be followed under Art. 10.5 of the ICN. However, when Nakai (1940) adopted *Theopsis* as a generic name, he did not exclude the type of Cohen-Stuart’s (1916: 70) sect. Theopsis because the section had not yet been typified, so Arts. 48.1 & 48.2 of the ICN do not apply. According to Art. 48.1 Note 1 of the ICN, this situation should be dealt with under Art. 7.3 of the ICN. Therefore, the genus Theopsis is typified by the type of its basionym, sect. Theopsis, that is, *C.cuspidata*, based on Chang’s (1981: 128) typification (Art. 10.5 of the ICN) even though the species was excluded from this genus (Art. 7.3 of the ICN).

However, *C.cuspidata* has nomenclatural problems. Kochs (1900: 586) described *T.cuspidata* Kochs and cited the single gathering *Henry 7026*. Subsequently, a name, *C.cuspidata*, was provided in the list of “Awards of Merit” in *The Gardeners’ Chronicle* (Anonymous 1912: 228). The plant was described as having “small, single, white flowers” with “pale-yellow stamens” and “narrow leaves” that were about 2–2.5 inches long. The brief description could make *C.cuspidata* validly published as a new species because the requirements of Arts. 32.1 & 38.1 of the ICN are likely fulfilled (also see Art. 38 Note 2 of the ICN). However, although Kochs’s *T.cuspidata* was neither directly nor indirectly referenced in the protologue of *C.cuspidata* (Anonymous 1912: 228; Arts. 41.1–41.3 of the ICN), the latter is, nevertheless, treated as a new combination based on *T.cuspidata* under Art. 41.4 (see Ex. 12) of the ICN.

Furthermore, two duplicates of *Henry 7026* were found at K and US, viz. K000380525 (the image is available at http://www.kew.org/herbcatimg/165067.jpg) and US 00504123 (the image is available at http://n2t.net/ark:/65665/308ec5722-d414-4a4d-b733-f34c3778997b). Since Kochs (1900: 586) did not indicate a single specimen of the entire gathering as the holotype, the two duplicates at different herbaria are syntypes of *T.cuspidata* based on Art. 40 Note 1. When Sealy (1958: 57) cited “*A. Henry 7026* (K, type-number)” under *C.cuspidata*, the citation could be treated as the lectotypification of the species following Arts. 7.11, 9.10 & 9.19 of the ICN. Therefore, the lectotype of *C.cuspidata* is that of *T.cuspidata*, viz. *Henry 7026* (K000380525), which, in turn, serves as the type of *Theopsis* (Table 1).

## Supplementary Material

XML Treatment for Camelliastrum

XML Treatment for Desmitus

XML Treatment for Salceda
